# Genetic and Epigenetic of Medullary Thyroid Cancer

**DOI:** 10.22034/ibj.22.3.142

**Published:** 2018-05

**Authors:** Fatemeh Khatami, Seyed Mohammad Tavangar

**Affiliations:** 1 Chronic Diseases Research Center, Endocrinology and Metabolism Population Sciences Institute, Tehran University of Medical Sciences, Tehran, Iran; 2 Department of Pathology, Dr. Shariati Hospital, Tehran University of Medical Sciences, Tehran, Iran

**Keywords:** Thyroid carcinoma, Genetic markers, Proto-oncogene

## Abstract

Medullary thyroid carcinoma (MTC) is an infrequent calcitonin-producing neuroendocrine tumor that initiates from the parafollicular C cells of the thyroid gland. Several genetic and epigenetic alterations are collaterally responsible for medullary thyroid carcinogenesis. In this review article, we shed light on all the genetic and epigenetic hallmarks of MTC. From the genetic perspective, *RET*, *HRAS*, and *KRAS* are the most important genes that are characterized in MTC. From the epigenetic perspective, Ras-association domain family member 1A, telomerase reverse transcriptase promoter methylations, overexpression of histone methyltransferases, *EZH2* and *SMYD3*, and wide ranging increase and decrease in non-coding RNAs can be responsible for medullary thyroid carcinogenesis.

## INTRODUCTION

Medullary thyroid carcinoma (MTC) is a rare neuroendocrine tumor that originates from the parafollicular cells (C cells) and produces calcitonin[1]. Approximately, a quarter of MTCs are genetic in nature; they are caused by a mutation in the rearranged during transfiction (RET) proto-oncogene, a receptor tyrosine kinase gene, which can undergo oncogenic activation through both cytogenetic rearrangement and activation of point mutation. REven though MTC is mostly sporadic (70-80%), some hereditary patterns can be seen in 20-30% of cases: these are classified as familial MTC (FMTC) with autosomal dominant trait[2-5]. High serum concentration of calcitonin and carcinoembryonic antigen is regularly regarded as MTC markers in blood[6-8]. It is common knowledge that cancer is the result of genetic changes accumulated in a manner that disturbs the normal homeostatic stability between cell proliferation and cell death[9,10]. In addition to genetic changes, epigenetic events have been considered as key indicators of carcinogenesis. Research on epigenetics has become gradually noticeable with the aim of understanding the role of epigenetic mechanisms in the abnormal events leading to cancer[11-13]. In fact, previous studies on cancer suggest that genetic and epigenetic alterations are two sides of the same coin responsible for morphological changes occurring during cancer progression[12,14-16]. Moreover, the notion that early-stage cancer is not as systematically aggressive as late-stage cancer is based on the finding that gene expression profiles is alike in early-stage cancer and fully metastatic cancer[17-19]. Thus, both genetic and epigenetic events correspond to several steps of carcinogenesis. In this review, we summarize current concepts on genetic and epigenetic changes associated with MTC and then discuss their potential relevance as biomarkers for cancer detection, diagnosis, and prognosis.

### Hallmarks of genetic MTC

A mutation is a stable modification in the DNA sequence of a given gene, which may alter the normal gene function[20]. Mutations can occur anywhere, from a single DNA building block (base pair) to a large segment of a chromosome, including multiple genes[20]. Some of the mutations are heritable, i.e. they are inherited from a parent and are present throughout a person’s life in virtually every cell in the body. These mutations are called germline mutations since they are present in the parent’s egg or sperm cells (germ cells)[20,21]. Other group of mutations are acquired mutations (or somatic): These happen only at a particular time during a person’s life and are present only in certain cells in the body[20]. These mutations can be caused by environmental factors, such as ultraviolet radiation from the sun or can occur if an error takes place in DNA replication during cell division. Acquired mutations in somatic cells (cells other than sperm and egg cells) cannot be passed to the next generation[22-25].

**Fig. 1:**
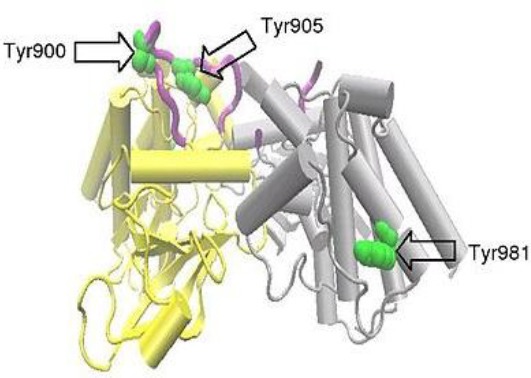
A RET dimer formed between two protein molecules, each spanning amino acids 703-1012 of the RET molecule and covering RET intracellular tyrosine kinase domain. One protein molecule, molecule A, is shown in yellow and the other, molecule B, in grey. The activation loop is colored purple and selected tyrosine residues in green. Part of the activation loop from molecule B is absent. mRET proto-oncogene with three main domains: an N-terminal extracellular domain with four cadherin-like repeats and a cysteine-rich region, a hydrophobic transmembrane domain, and a cytoplasmic tyrosine kinase domain. Phosphorylation of Tyr981 and the additional tyrosinesTyr1015, Tyr1062, and Tyr1096 are not covered by the above structure, though these have been shown to be important for initiation of the intracellular signal transduction processes[36].

MTC has been described in two forms: sporadic and hereditary/familial. About one-fourth of MTC patients haveone of three different syndromes, which are FMTC, multiple endocrine neoplasia type 2A (MEN 2A), or type 2B (MEN 2B). Around a half of the patients with MEN 2A or MEN 2B develop pheochromocytomas[26-30]. Moreover, 25% of patients with MEN 2A will possibly develop primary hyperparathyroidism[28,31], while patients with MEN 2B develop marfanoid habitus and mucosal/intestinal ganglioneuromatosis[32]; patients with just FMTC individually develop MTC[29,32]. In fact proto-oncogene RET germline mutations is presented in 90% of patients with hereditary MTC (FMTC, MEN 2A, or MEN 2B)[33]. Thus, the entire hereditary syndromes are attributed to the same disease-causing gene[34,35]. *RET* proto-oncogene is a tyrosine kinas receptor coding gene, and it is an element of the glial cell line-derived neurotrophic factor (*GDNF*) family which are classified as extracellular signaling molecules[36]. Human *RET* gene with 21 exons is localized on chromosome 10 (10q11.2)[37,38]. Like other tyrosine kinase receptors, RET is able to motivate several signaling pathways, including RAS/extracellular signal-regulated kinase (*ERK*), phosphatidylinositol 3-kinase (*PI3K*)*/AKT*, p38 mitogen-activated protein kinase (*MAPK*), and c-Jun N-terminal kinase (*JNK*) pathways[30,39-44]. The typical splicing of the *RET* gene results in three different isoforms. The C-terminal region in *RET51, RET43*, and *RET9* have 51, 43, and 9 amino acids, respectively[45]. As shown in Figure 1—which is premised on the protein data bank code 2IVT—all RET protein isoforms can be subdivided into three main domains: an N-terminal extracellular domain with four cadherin-like repeats and a cysteine-rich region, a hydrophobic transmembrane domain, and a cytoplasmic tyrosine kinase domain that is divided through the 27 amino acids insertion[36,46].

**Fig. 2:**
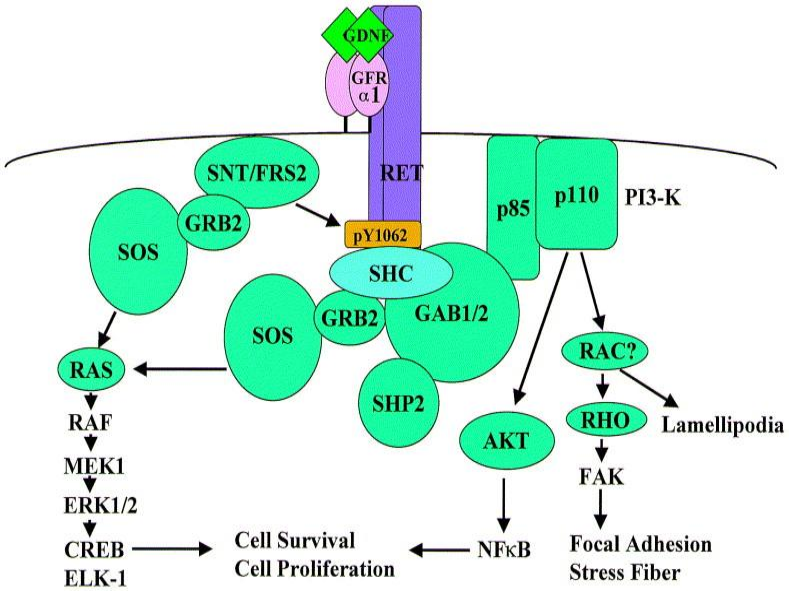
Intracellular signaling pathways mediated by activated RET[55].

Glial cell line-derived neurotrophic factor and some other related molecules like neurturin, artemin, and persephin trigger an intracellular signaling pathway through a unique multi-component receptor systems including glycosyl-phosphatidylinositol-anchored co-receptor in addition to *RET* tyrosine kinase[37,40,47-49]. These neurotrophic factors support the survival of many neurons, including the central motor dopamine neurons; they also cause peripheral autonomy [50-52] more than renal development and facilitate regulation of spermatogonia differentiation (Fig. 2)[53,54].

**Fig. 3:**
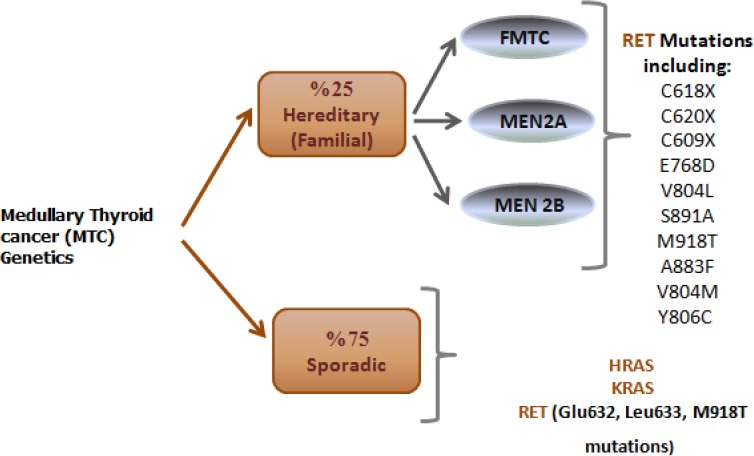
Genetics of medullary thyroid cancer.

Unfortunately, unlike hereditary MTC, the etiology of sporadic MTC has not been completely elucidated. This is despite the fact that 75% of all MTC patients being sporadic without any family history of MTC or incidence of any other MEN 2-specific disease. Recently, some additional mutations, such as *HRAS* and *KRAS* mutations, have been found to be more appropriate diagnostic markers than *RET* for MTC. This observation has been suggested on the basis of high-throughput mutation profiling study (Fig. 3)[56-59]. In few cases of sporadic MTC, a deletion of codons Glu632 and Leu633 of *RET* proto-oncogene was identified. These mutations activate the *RET* gene more effectively than the Cys634Arg missense mutation. They also induce stable dimer formation in the absence of ligand[60,61].

In more aggressive phenotypes of sporadic MTC, the *M918 TRET* mutation has been found in around 30 to 50% of cases[62]. Actually, the somatic *RET* mutation (*M918T*) is associated with stage of the disease and persistence of the disease after total thyroidectomy because it makes the chance of recurrence and metastasis greater than before and reduces chances of free survival[62,63]. A connection between the presence of this somatic mutation with the more advanced pathological TNM stage has also been identified[64-67].

### Epigenetic hallmarks of MTC

**Fig. 4:**
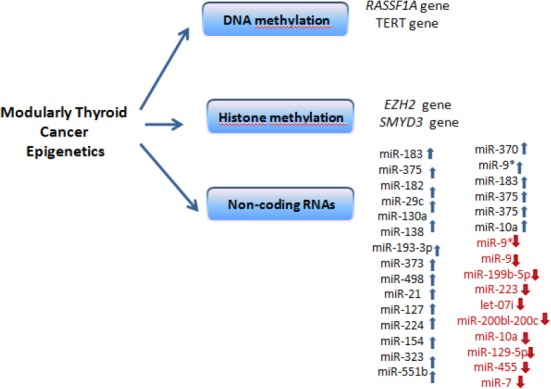
Epigenetics of medullary thyroid cancer.

The word ‘epigenetics’ refers to covalent modification of DNA, protein, or RNA, resulting in changes in the function and/or regulation of DNA without modification of their original sequences. In some cases, epigenetic modifications can be stable and can pass on to future generations; mostly, however, they are vigorous modifications in response to environmental stimuli[68]. The major mechanisms responsible for epigenetic regulation are DNA methylation, histone modifications, and non-coding RNAs[59,69-72]. The role of epigenetics in MTC is largely defined as hypermethylation of CpG islands in the promoter region of Ras-association domain family member 1A (*RASSF1A*)[73,74] and telomerase reverse transcriptase (*TERT*) genes[75], overexpression of histone methyltransferases like *EZH2* and *SMYD3*[76,77], and microRNAs (miRNAs) expression profile (Fig. 4)[78-84].

In spite of the fact that *RASSF1A* gene promoter hypermethylation is linked to more aggressive thyroid cancers, CpG island methylation of tumor-associated genes— such as *p16, TSHR, MGMT*, and *PTEN*—have not shown any significant degree of hypermethylation in MTC[73,85-87]. Nevertheless, the existence of methylation in the promoter region of *TERT* gene and consequent variation of DNA copy numbers within a huge cohort study of MTC cases have been documented[75]. In fact, telomerase is a protein responsible for keeping and fixing telomeres of the chromosomes. Its activation by *TERT* has been increased frequently in many types of cancers, including MTC[86]. Wang and his colleagues[75] have reported that *TERT* gene hypermethylation is related to high DNA copy number, and MTC patients with higher *TERT* methylation have lower chances of survival[75].

Epigenetic control through histone methyltrasferases in more aggressive forms of MTC have recently been investigated by Sponziello *et al*.[76]. In fact, the platform of epigenetic regulatory factors and their mRNA levels profiling in a big cohort of MTC tissues has revealed the fact that overexpression of two histone methyltransferases, *EZH2* and *SMYD3*, is connected with higher risk of metastases, disease consistency, and finally death of patients: These can be prognostic biomarkers for MTC[76,88]. Remarkably, gene expression profile was free of *RET* or *RAS* mutations.

The most common mutation is related to the *RET* mutation in genetic alteration of MTC tumorigenesis. The transcriptional activity of *RET* is controlled by epigenetic processes as well. It has been demonstrated that in colorectal cancer CpGisland methylation of *RET* gene promoter is a potential prognostic marker for stage II of the disease[89,90]. Patients with considerable hypermethylated *RET* have worse overall survival compared to those with unmethylated RET promoter[91]. Significantly, *RET* expression is regulated by a transcription factor, homeobox B5[92], which is related to the multi-species conserved sequence in the primary intron of the *RET* gene in addition to the higher level of *RET* transcription. Another regulating mechanism of RET transcription level is acethylation because in human neuroblastoma cells with a low *RET* mRNA level, histone deacetylase inhibitor and sodium butyrate cause hyper acetylation and increase the transcription of *RET* gene[93].

MiRNAs are small non-coding RNAs with the lengths of 20-23 nucleotides; they are classified as epigenetic modifiers[94]. MiRNAs are non-protein-coding RNAs that alter gene expression through mRNA translation inhibition or by means of the target molecule degrading[94,95]. In reality, mutations or abnormal expression of miRNAs are more often related to the pathogenesis of a wide range of cancers because they affect both tumor suppressors and oncogenes[96]. In spite of the fact that several studies have highlighted the role of miRNA profiling of MTC and its malignancy (Table 1), due to difficulty in obtaining normal C cells, none of the existing literature has compared miRNA profiles between MTC and normal C cells[97].

**Table 1:** A list of suggesting microRNA (miRNA) in medullary thyroid cancer

A signature of increased and decreased miRNA associated with MTC	
Increasing miRNA	miR-183, miR-375, miR-182, miR-29c, miR-130a, miR-138, miR-193-3p, miR-373, miR-498, miR-21, miR-127, miR-224, miR-154, miR-323, miR-551b, miR-370, miR-9, miR-183, miR-375, miR-375, miR-10a
Decreasing miRNA	miR-199b-5p, miR-223, let-07i, miR-200bl-200c, miR-10a, miR-129-5p, miR-455, and miR-7, miR9

Ten miRNAs were shown to have different expression pattern between sporadic MTC and

hereditary MTC[78]. In correlation with clinical outcomes, high levels of *miR-183* and *miR-375* were linked to the lateral lymph node and distant metastases[78]. Significantly, similarity in miRNA profiles of *miR-183*, *miR-375*, and *miR-9-3p* (*miR-9*) between primary tumor tissues and lymph node metastasis tissues was observed[79]. This was further to the role of *miR-9-3pin* in regulation of autophagy[80]. More than that, a comparison of miRNA profiling between primary and metastatic forms of MTC highlighted 10 deregulated miRNAs[81-83,98]. There is possibility that the constitutive activation of RET, as a crucial occurrence in MTC tumor genesis, is regulated through epigenetic mechanisms like miRNAs[81,99,100].

From genetic point of view, *RET* mutations in codons *609* (*C609X*), *618* (*C618X*), *620* (*C620X*), *786* (*E768D*), *804* (*V804L*), *819* (*S891A*), *918* (*M918T*), *833* (*A883F*), *804* (*V804M*), *806* (*Y806C*), *632* (*Glu632*)*, 633* (*Leu633*)*, and 918 (M918T*) as well as *HRAS*, and *KRAS* mutations are the most important mutations that cause medullary thyroid carcinogenesis. From epigenetic perspective, *RASSF1, TERT* promoter methylations, histone methyltransferases (*EZH2* and *SMYD3*) overexpression, and wide ranging increase and decrease of non-coding RNAs contribute to medullary thyroid carcinogenesis.
